# Multi-level analysis of the determinants of physical domestic violence against children using longitudinal data from MINIMat mother–child cohort in Bangladesh

**DOI:** 10.3389/fpubh.2023.1185130

**Published:** 2023-12-29

**Authors:** Ruchira Tabassum Naved, Jannatul Ferdous Antu, Kausar Parvin, Shirin Ziaei

**Affiliations:** ^1^Gender, Equity and Rights Research Group, Health Systems and Population Studies Division, icddr,b, Dhaka, Bangladesh; ^2^Unit of Occupational Medicine, Institute of Environmental Medicine, Karolinska Institutet, Stockholm, Sweden

**Keywords:** violence against children, violence against mother, multi-level analysis, rural Bangladesh, gender

## Abstract

**Objectives:**

Despite high levels of physical violence against children (VAC) globally (40–50%), the literature on the determinants of VAC remains inconclusive. Most of the literature on this topic is based on cross-sectional data, and the multi-level nature of the drivers of VAC is widely ignored. This leads to model specification problems and an inability to draw causal inferences. Moreover, despite the higher prevalence of VAC in low-and middle-income countries, studies from high income countries dominate the field. We examined the determinants of physical domestic VAC to address these gaps in the literature.

**Methods:**

Data were collected between 2001 and 2020 from 762 mother–child dyads recruited in the Maternal and Infant Nutrition Interventions in Matlab (MINIMat) study in Bangladesh. We conducted multi-level logistic regression analyses to identify the determinants of physical domestic VAC.

**Results:**

Prevalence of physical domestic violence against girls (69%) and boys (62%) was extremely high. Community-level prevalence of physical domestic VAC increased the likelihood of physical domestic VAC at the individual level across gender (girls - OR-5.66; 95% CI- 3.11-10.32; boys - OR-7.67; CI- 3.95-14.91). While physical domestic violence against mothers was not associated with physical domestic violence against girls, it reduced the likelihood of such violence against boys by 47%. Having 3 or more siblings predicted physical domestic violence against girls (OR-1.97; 95% CI- 1.01-3.81 for 3 siblings; OR-4.58; 95% CI- 2.12-9.90 for 4 or more siblings), but not against boys. While girls in Hindu families were more likely to experience this violence, the boys were not. Mother’s education, employment non-governmental organization (NGO) participation and, household wealth did not predict this violence against any gender.

**Conclusion:**

We contend that physical domestic violence against mothers reflects an emphasized patriarchal culture in a family where a boy is less likely to experience physical domestic violence. Social norms and social learning theories explain the greater likelihood of a child experiencing physical domestic violence in a village with a higher level of such violence. We conclude that social norms around physical domestic VAC and patriarchal culture need to be changed to effectively address this violence.

## 1 Introduction

Violence against children (VAC) is a global public health, human rights, and development issue. Globally, 50% of children aged 2–17 experience violence (1). According to UNICEF, around 63% of children ages 2–14 are regularly exposed to physical violence by their caregivers (2). Another systematic review representing 171 countries reports that between 40 and 50% of girls and boys aged 2–14 experienced physical violence in the past month by a caregiver or household member (3). Overall, a higher proportion of boys reported experiencing physical violence than girls (4).

According to the literature, factors commonly associated with VAC are age, sex, mothers’ experiences of violence, the mental health of the perpetrator, childhood trauma of the perpetrator, household poverty, and food insecurity (5–10). Although an ecological framework is widely acknowledged to explain VAC (11–15), appropriate analytical methods are often not used to identify the determinants of VAC. Evidence suggesting a clustering of VAC at the community level with rigid social and gender norms endorsing violence and gender inequality significantly contributing to VAC (5–7, 16, 17). Unfortunately, studies often ignore such broader social context (18).

Low- and middle-income countries (LMIC) report relatively higher prevalence of VAC compared to high-income countries (HIC). Most of the literature on this topic, however, comes from the latter (19). According to the nationally representative Multiple Indicator Cluster Survey (MICS) survey conducted in 2019, VAC is pervasive in Bangladesh with 65% of the children aged 1–14 years being ever exposed to physical violence (20). These high rates were accompanied by 35% of the caretaker sample holding the belief that physical punishment is essential for component of child rearing (20). In a study conducted by Mamun et al. in 2022, one in two parents of 10 to 19-year-old children endorsed child beating (21).

According to the Bangladesh Adolescent Health and Well-Being Survey (22) the pattern of physical violence against adolescents is gendered, with a higher proportion of boys reporting it during the last 12 months compared to girls (26% vs. 20%). The same source reports that physical violence against girls was most commonly perpetrated by family members, while the main perpetrators of physical violence against boys were their peers, followed by family members.

There is a paucity of literature on the determinants of physical domestic violence against children in Bangladesh. To our knowledge, the studies exploring correlates of physical VAC in Bangladesh included all perpetrators, regardless of their relationship with the child (23) despite the fact that the drivers of domestic physical violence against children are not likely to be exactly the same as those driving VAC by other perpetrators. These studies recognize that gender is a potential contributor to VAC and thus include gender as an independent variable in the model. This, however, is not enough for identifying correlates of VAC against boys and girls, which are likely to be different. Another serious limitation of these studies is that multi-level modeling appropriate for identifying determinants of outcomes explained by ecological conceptual framework was not used in any of them. Further, these studies were based on cross-sectional data, which inhibited drawing any causal inference. We attempt to address these gaps in the literature by examining the determinants of physical domestic violence against boys and girls using multi-level logistic regression analysis of longitudinal data collected between 2001 and 2020 as part of the Maternal and Infant Nutrition Interventions in Matlab (MINIMat) study conducted in the south-east of Bangladesh.

## 2 Methods

### 2.1 Study setting, design, and participants

This study was embedded in a larger longitudinal study well-known as the MINIMat trial (Maternal and infant nutrition interventions, reg#ISRCTN16581394). The details of the study are described elsewhere (24). Briefly, the MINIMat trial is a population-based food and micronutrient supplementation trial for pregnant women. The trial was conducted in Matlab, a predominantly rural sub-district of Bangladesh, where icddr,b (an international research organization based in Bangladesh), has been running a Health and Demographic Surveillance System (HDSS) since 1966. From November 2001 to October 2003, all pregnant women from the HDSS area were invited to participate in the MINIMat trial. The enrolled pregnant women (*n* = 4,436) were randomized into two types of food and three types of micronutrient supplementation groups following a two-by-three factorial design. Women were interviewed monthly at home and in the clinics at 14, 19, and 30 weeks of gestation. After delivery of the index child, the mother–child dyads were followed up relatively intensively for two years and later with a greater interval. This analysis includes data collected during pregnancy and at 10- and 18-year follow ups.

**Figure 1:**
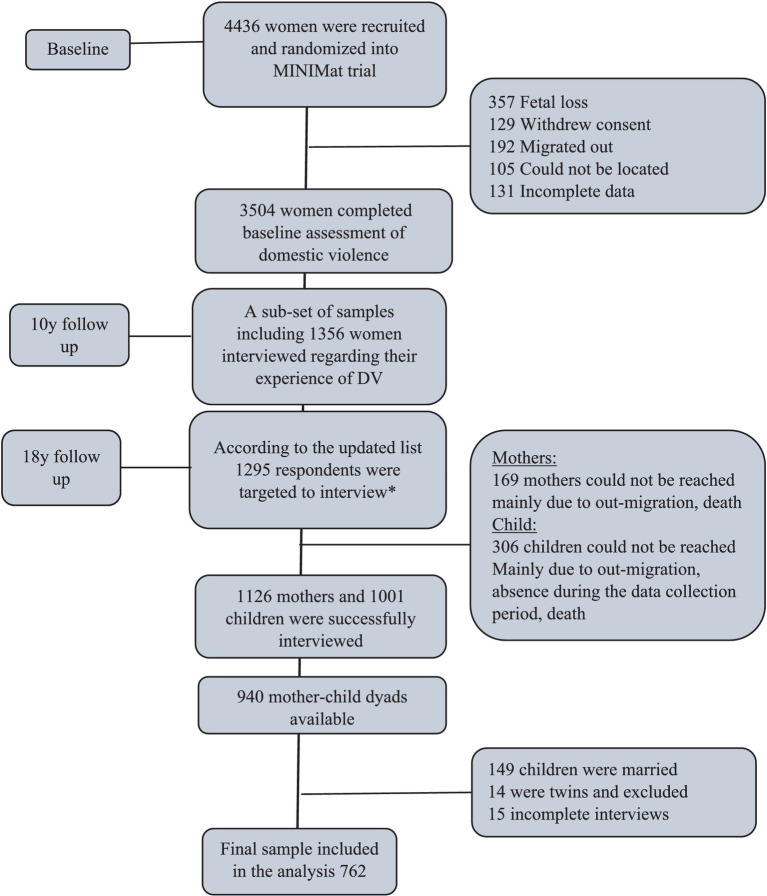
CONSORT flow diagram of the number of MINIMat Mother and children in three rounds of data collection between 2001 and 2020. *The list of MINIMat mother and children were updated at 14 years.

Socio-demographic data were collected from the mothers during a household visit at enrolment as well as in follow up interviews. A team of trained paramedics interviewed women at the clinic during the 30^th^ week of gestation regarding their experience of domestic violence (DV). Among the recruited pregnant women, 3,504 completed the DV assessment (Figure 1). The main reasons for loss to follow up were: fetal loss, out-migration, and withdrawal of consent to participate in the study.

Only mothers whose children were born between April 2002 and June 2003, representing a one calendar year birth cohort, were invited to participate in the 10-year follow-up interviews (*n* = 1,356) (25). Women were interviewed again regarding their experience of DV during the interim period, using the same standard questionnaire. The women who completed the DV module during pregnancy and the 10-year follow-up were approached for an interview in the 18-year follow-up conducted during 2020–2021. Among them, 1,126 women were successfully interviewed.

The survey of the index children at the 18-year follow up included a module on VAC. A total of 1,001 children completed the interview, resulting in a total of 940 mother–child dyads for this analysis. Since the experience of violence radically differed between married and unmarried children (26), this analysis focuses only on unmarried children. Only singletons were included in the analysis. Thus, we derived a total of 762 mother–child dyads for our analyses, with 422 male and 340 female children (Figure 1).

### 2.2 Measures

#### 2.2.1 Outcome variables

The outcome variable was lifetime exposure to physical domestic violence among girls and boys. Physical domestic VAC was measured using the 17-item International Society for the Prevention of Child Abuse and Neglect (ISPCAN) Child Abuse Screening Tools (ICAST) (27). Examples of items include slapping, kicking, pulling hair, twisting ears etc. We validated this scale using exploratory factor analysis (EFA). We performed Q-type EFA, which calculates the factors from the individual responses.

**Figure 2:**
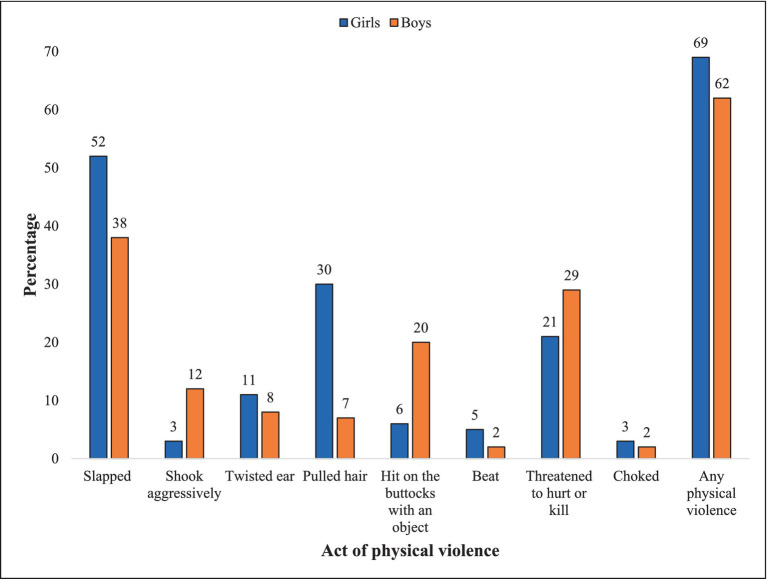
Exposure to lifetime physical domestic violence by act and sex (Boys, *N* = 422; Girls, *N* = 340).

The validated scale retained eight items (e.g., slapping, beating) (See Figure 2) (Cronbach’s alpha = 0.70 and KMO = 0.80). A positive response to any of these items was considered as indicating that the child was exposed to lifetime physical domestic violence and was coded as ‘1 = Yes’, otherwise as ‘0 = No’.

#### 2.2.2 Exposures

In selecting the exposure variables, we considered previous literature, the availability of relevant MINIMat data, and significant bivariate associations between the outcome and exposure variables. To ensure temporality, to the extent possible, we leveraged the longitudinal nature of the data and used lagged variables as covariates. Where such data from previous time point/s were not available we used time invariant exposure variables. We used the measurements that had the lowest missing values and inconsistencies at the three time points. In this paper, we refer to the survey during pregnancy as T1, the 10-year follow up as T2, and the 18-year follow up as T3. The number of siblings (including he/she) of a child (T2) was coded as ‘1’, if she had 1–2 siblings; ‘2’, if she had 3 siblings; and ‘3, if, she had 4 or more siblings’.

##### 2.2.2.1 Mother’s characteristics

Mother’s education at T3 was coded as, ‘1’ for no education; ‘2’ for 1–5 years of education; ‘3’ for ‘6–10 years of education; and ‘4’ for more than ‘>10 years of education. In the context of Bangladesh, particularly in rural areas, female education usually stops with marriage. Since the education of a woman is usually time invariant, we used it as a proxy for education at an earlier time point in adulthood.

A mother not being employed at T1 was coded as ‘0’ and ‘1’ otherwise. Not participating in any Non-governmental organization (NGO) at T2 was coded as ‘1’; participation in the microcredit program only as ‘2’; participation in other types of NGO as ‘3’; participation in both types of NGOs as ‘4’.

A modified version of the conflict tactic scale (28) was used to measure the mother’s lifetime experience of physical DV (T1). A total of seven items (e.g., slapping, kicking, choking, or burning) were used to measure physical DV. A woman responding positively to any of these questions was treated as exposed to physical DV and coded as ‘1’, otherwise as ‘0.’

##### 2.2.2.2 Household characteristics

An extended household at T1 was coded as ‘1’ and a nuclear household as ‘0’. Household wealth quintiles at T1 were derived by dividing the household asset scores obtained from principal component analysis into five categories. The categories were as follows: poor (1), lower middle (2), middle (3), upper middle (4), and rich (5). Families pursuing Islam at T3 were coded as ‘0’ and Hinduism as ‘1’.

##### 2.2.2.3 Community characteristics

The prevalence rate of physical domestic violence against girls and boys at the community-level at T3 was calculated separately for boys and girls using the same procedure. First, the number of individuals exposed to physical domestic violence in a village was calculated, divided by the sample size in the village, and then multiplied by 100. For 31–40% of the villages, the rate of community-level physical domestic VAC was more than 75%. We have coded villages with such high rates of physical domestic VAC as ‘1’ and otherwise as ‘0’.

### 2.3 Statistical analyses

Descriptive analyses were performed to describe the background characteristics of the study participants and the prevalence of physical domestic VAC. We examined differences between the background characteristics of girls and boys using chi-square tests for categorical variables and t-tests for continuous variables. Multi-level regression analyses were conducted separately for the boy and girl samples. At first, a null model (Model 1) was developed to estimate the community level variance to justify using the multi-level logistic regression model. The intra-cluster correlation (ICC) value was estimated at 0.18 and 0.03 for boys and girls, respectively, implying that community-level factors can explain 18% for boys and 3% for girls of the total variation in physical domestic VAC. Second, in Model 2, individual-level factors were incorporated. Finally, in model 3, community level variables were included. All the analyses were performed using STATA version 15, and the significance level for all statistical tests was set at 5%.

### 2.4 Ethical considerations

All rounds of the MINIMat trial (PR-2000-025, PR-12022, and PR-19101) were approved by icddr,b’s institutional review board. The third round was additionally approved by Swedish Research Ethics Authority (# 2021–00523).

## 3 Results

### 3.1 Characteristics of the study sample

Table 1 shows the characteristics of the study participants by sex. The mean age for both sexes was 17.5 years. Around 20% of the mothers had no education, and only 8% had education beyond ten years. The number of siblings was significantly higher among mothers of girls compared to those of boys. About 8% of the mothers were employed. Regardless of the sex of the index child, approximately, 66% of the mothers were NGO members. A significantly higher proportion of boys’ mothers reported lifetime physical DV when interviewed in the pregnancy with the index child, compared to the mothers of girls (25% vs. 17%). About 38% of the mothers came from a nuclear family at T1. Household socio-economic status was significantly different for the boys and the girls with a higher proportion of girls coming from better off families. The samples were predominantly Muslim (85%). A higher proportion of the girls (40%) came from a community with high prevalence of physical domestic violence against girls (i.e., > = 75%), compared to proportion of boys (31%) living in a community with high prevalence of physical domestic violence against boys.

**Table 1:** Background Characteristics of the sample by child sex, *N* = 762.

	All sample, % (*n*) (*N* = 762)	Girls, % (*n*) (*N* = 340)	Boys, % (*n*) (*N* = 422)	*p*-value*
Child characteristics				
Mean age (SD, range)	17.46 (0.51, 16–18)	17.46 (0.52, 16–18)	17.46 (0.51, 16–18)	0.968
Lifetime exposure of children to physical domestic VAC	64.83 (494)	68.53 (233)	61.85 (261)	0.055
Number of siblings				
1–2	31.50 (240)	29.41 (100)	33.18 (140)	
3	39.76 (303)	35.00 (119)	43.60 (184)	0.001
4 and above	28.74 (219)	35.59 (121)	23.22 (98)	
**Mother’s characteristics**				
Mother’s Education				
No education	20.21 (154)	20.88 (71)	19.67 (83)	
1–5 years	34.91 (266)	32.94 (112)	36.49 (154)	0.790
6–10 years	37.14 (283)	38.24 (130)	36.26 (153)	
11–12 years	7.74 (59)	7.94 (27)	7.58 (32)	
Mother’s employment status				
Yes	8.01 (61)	6.18 (21)	9.48 (40)	0.095
No	91.9 (701)	93.82 (319)	90.52 (382)	
NGO membership				
None	33.86 (258)	34.41 (117)	33.41 (141)	
Micro-credit only	19.29 (147)	17.06 (58)	21.09 (89)	0.451
Other NGO	28.22 (215)	30.29 (103)	26.54 (112)	
Both	18.64 (142)	18.24 (62)	18.96 (80)	
Mother’s exposure to physical DV	21.39 (163)	16.76 (57)	25.12 (106)	0.005
**Household characteristics**				
Family structure, Nuclear	38.19 (291)	36.47 (124)	39.57 (167)	0.381
Wealth index				
Poor	20.21 (154)	20.00 (68)	20.38 (86)	
Lower middle	22.31 (170)	20.29 (69)	23.93 (101)	
Middle	23.75 (181)	22.65 (77)	24.64 (104)	0.022
Upper middle	17.19 (131)	15.59 (53)	18.48 (78)	
Rich	16.54 (126)	21.47 (73)	12.56 (53)	
**Religion**				
Muslim	85.04 (648)	85.00 (289)	85.07 (359)	0.978
Hindu	14.96 (114)	15.00 (51)	14.93 (63)	
**Community-level characteristics**				
Rate of physical domestic VAC >75%, %	35.17 (268)	40.00 (136)	31.28 (132)	0.012

*p-values are based on a t-test for continuous variables and χ2 tests for categorical variables, comparing frequencies of variables by gender.

As shown in Figure 2, the prevalence of lifetime physical domestic violence was 69% among the girls and 63% among the boys. Slapping was the most common act of physical domestic violence across sexes (38–52%) and choking – the least common (2–3%). A higher proportion of girls experienced all moderate acts of physical domestic VAC (e.g., such as slapping, shaking, ear and hair pulling). Exposure to beatings was more common among girls than boys. The two acts to which boys were more exposed to than the girls were spanking (20% vs. 6%) and experiencing threats to hurt or kill them (29% vs. 21%).

### 3.2 The determinants of lifetime physical domestic violence against children

Table 2 presents the results of the multi-level logistic regression analyses of the determinants of physical domestic VAC by sex. Judging by the size of the Akaike information criterion (AIC) in the three sets of models run for each sex, it is evident that the Model 3, where both individual/household-and community-level factors were included, shows the best fit for both girl and boy samples. The ICC in the final model for the girls was reduced from 0.08 in model 2 to 1.80e-34 which implied that the prevalence of physical domestic violence against girls in the community explained almost all the community-level variations in the physical domestic violence among girls. The ICC values in the three sets of models run on the boys’ sample (0.18 in Model 2 vs. 0.04 in Model 3) showed that in Model 3, 14% of the community-level variations in physical domestic violence against boys could be explained by the community-level prevalence of physical domestic violence against them. Clearly, Model 3 provided the best estimates of the determinants of physical domestic VAC for each sex.

**Table 2:** Determinants of lifetime physical domestic violence against children aged 16–18 years: results from the multi-level logistic regression models, *N* = 762.

	Girls (*N* = 340)			Boys (*N* = 422)		
Variables	Model 1 (Null model)	Model 2 (Null + individual/household level covariates)	Model 3 (Null + individual/household+ village level covariates)	Model 1 (Null model)	Model 2 (Null + individual/household level covariates)	Model 3 (Null + individual/household+ village level covariates)
	OR (95% CI)	OR (95% CI)	OR (95% CI)	OR (95% CI)	OR (95% CI)	OR (95% CI)
Individual/household level variables						
Mother’s Education (T3)						
No education (ref)						
1–5 years		1.37 (0.63–2.98)	1.32 (0.60–2.90)		1.75 (0.92–3.32)	(0.83–2.96)
6–10 years		2.04 (0.82–5.08)	2.15 (0.86–5.41)		1.19 (0.58–2.43)	1.01 (0.49–2.05)
11–12 years		2.86 (0.71–11.50)	2.66 (0.63–11.17)		2.23 (0.63–6.51)	1.71 (0.55–5.33)
Number of siblings (T2)						
1–2 (ref)						
3		1.85 (0.97–3.53)	1.97 (1.01–3.81)*		1.65 (0.95–2.85)	1.61 (0.94–2.78)
4 or more		4.18 (1.94–8.99)*	4.58 (2.12–9.90)*		2.08 (1.04–4.17)*	1.85 (0.93–3.67)
Mother’s employment status (T1)						
No (ref)						
Yes		0.90 (0.30–2.67)	1.01 (0.34–3.00)		1.61 (0.70–3.68)	1.54 (0.68–3.46)
Mother’s NGO participation (T2)						
None (ref)						
Microcredit program only		1.67 (0.75–3.72)	1.44 (0.65–3.18)		0.75 (0.39–1.43)	0.60 (0.31–1.16)
Other NGO		0.97 (0.43–1.76)	0.95 (0.49–1.82)		1.14 (0.62–2.08)	1.04 (0.58–1.88)
Both		1.39 (0.65–2.98)	1.36 (0.63–2.91)		0.67 (0.35–1.29)	0.58 (0.30–1.12)
Lifetime physical domestic violence against mothers (T1)						
No (ref)						
Yes		0.87 (0.42–1.76)	0.82 (0.40–1.68)		0.48 (0.28–0.84)*	0.53 (0.31–0.92)*
Family structure (T1)						
Nuclear (ref)						
Extended		0.89 (0.49–1.59)	0.80 (0.44–1.44)		0.59 (0.36–0.96)*	0.54 (0.33–0.89)*
Wealth index (T1)						
Poor (ref)						
Lower middle		1.80 (0.77–4.25)	1.90 (0.80–4.51)		0.69 (0.34–1.38)	0.75 (0.38–1.50)
Middle		2.17 (0.88–5.30)	2.11 (0.86–5.22)		1.03 (0.49–2.16)	1.20 (0.57–2.51)
Upper middle		1.10 (0.41–2.99)	1.04 (0.38–2.83)		0.94 (0.41–2.15)	1.03 (0.45–2.34)
Rich		1.25 (0.44–3.56)	1.14 (0.39–3.30)		1.17 (0.45–3.05)	1.20 (0.46–3.12)
Religion (T3)						
Muslim (ref)						
Hindu		3.51 (1.36–9.04)*	2.99 (1.25–7.20)*		0.97 (0.46–2.08)	1.10 (0.54–2.21)
Community level variable						
Prevalence of physical domestic VAC (T3)						
Lower rates (ref)						
Higher rates			5.66 (3.11–10.32)*			7.67 (3.95–14.91)*
Random effect						
Estimate (Village level variation)	0.09	0.29	5.92e-34	0.72	0.70	0.14
ICC	0.03	0.08	1.80e-34	0.18	0.18	0.04
Intercept	2.21 (1.71–2.86) *	0.38 (0.13–1.11)	0.21 (0.07–0.60)*	1.70 (1.23–2.35)*	0.96 (0.37–2.49)	0.59 (0.23–1.53)
Model statistic						
AIC	427.02	429.10	393.21	546.47	554.81	516.36

*p < 0.05.

Model 3 shows that some household-level factors also predicted physical domestic violence against boys and girls. Thus, the risks of physical domestic violence increased with the number of siblings in the girl sample. Thus, compared to the girls who had 1–2 siblings, the girls who had three, or four or more siblings were more likely to experience physical domestic violence (OR-1.97; 95% CI- 1.01-3.81 in case of 3 siblings; OR-4.58; 95% CI- 2.12-9.90 in case of 4 or more siblings). The number of siblings had no effect, however, on the boys’ exposure to this violence. Mother’s experience of lifetime physical DV up to pregnancy with the index child did not affect girls’ exposure to this violence, while it reduced the risk of physical domestic violence among boys by 47% (OR-0.53; 95% CI- 0.31-0.92). Living in an extended family decreased the risks of physical domestic violence among boys 46% (OR-0.54; 95% CI- 0.33-0.89), while it did not affect the girls. Girls from Hindu families were at three times higher risk of being physically abused by family members compared to their Muslim counterparts (OR-2.99; 95% CI- 1.25–7.20). Religion, did not have any impact on the boy’s exposure to physical domestic violence.

In communities where the prevalence of physical domestic violence was 75% or more among girls, the likelihood of physical domestic violence was six times higher among girls (OR-5.66; 95% CI- 3.11-10.32). It was eight times higher among boys (OR-7.67; 95% CI- 3.95-14.91) in communities with 75% or higher prevalence among boys compared to communities with a lower prevalence rate.

## 4 Discussion

Our findings show higher prevalence of physical domestic violence among boys and girls in this sample (65%) compared to many other countries (9, 29). While the prevalence of physical domestic violence among boys is commonly reported to be higher than among girls (5), our findings show the opposite picture. We argue that this is not surprising given the patriarchal setting characterized by strong son preference and male privilege (30).

Our findings offer a deeper insight into the predictors of physical domestic violence against children by fitting separate models for boys and girls and by performing multi-level analyses. Thus, while the previous literature suggests that large family size (31) and greater number of siblings (32) increase the likelihood of VAC, our findings show that having a higher number of siblings increased the likelihood of physical domestic violence among girls, but not among boys. While the first may be due to increased stress on household resources and particularly on the mother’s time in juggling household responsibilities, the latter may highlight the privileged position of a son.

A study conducted in Agartala, India by Deb & Modak suggests that extended family protects children against physical domestic violence (33). This, however, was not substantiated by another study conducted in Jammu, India (34). Our findings are more nuanced and show that the extended family protected the boys against physical domestic violence, but not the girls. This may be explained by the following. Marriages are patrilocal in Bangladesh. When a female gets married, she usually joins an extended marital family. Eventually most of the extended families split to form nuclear families (35). It is plausible that representative/s of the older generation in an extended household hold great power, and at the same time they may hold more tightly on to patriarchal ideologies and practices that tend to protect boys from being physically abused by family members, but not girls. Differences in findings from different settings may suggest importance of the contextual differences. More importantly, our findings clearly show that the same factor may have differential effect on physical domestic violence against different genders and thus, results of analyses pooling both genders might may mask a different reality.

In contrast to many studies conducted both in developed and developing countries, poverty (6, 18, 36) and maternal education did not come out as predictors of physical domestic VAC in our study. This may indicate that this violence actually cuts across all households and all maternal education categories in this low educated patriarchal context dominated by age and gender hierarchies.

The finding that the Hindu girls were at higher risk of physical domestic violence compared to Muslims may be explained by the fact that as a minority group, Hindu families may face greater challenges in protecting the girls’ chastity linked family honour. Thus, they may be more likely to subject the girls to physical abuse for the purpose of controlling and disciplining them (37).

The literature presents compelling evidence on the intersections between violence against women and VAC (6, 37). Our findings are, however, nuanced and support the existing literature only partially. In contrast to the previous literature that suggests that violence against women increases the likelihood of VAC (38, 39), we have found an effect of violence against mothers on physical domestic violence among boys, but not among girls. Moreover, the relationship between the two found in this study contradicts the literature. Thus, violence against the mother in a family actually reduced physical domestic violence among boys. Our findings suggest that the nature of interactions between physical domestic violence against mothers and domestic VAC may be context specific and may not go in the same direction across settings. We argue that families where women are physically abused, practice emphasized patriarchy. Thus, in these families, sons were more privileged and, accordingly, were protected against physical domestic violence. The likelihood of physical abuse of girls in such families did not increase, but neither did it reduce as in case of the boys. Thus, it is important to underline that physical domestic violence against mothers is embedded in gender inequality, which in turn generates greater gender inequality in how male and female children are treated in the family.

Our results underline the importance of multi-level modeling of determinants of physical domestic VAC showing that almost one-fifth of the variations were explained by the community-level factors among the boys. This echoes claims made by other researchers (10). We find that community-level rates of physical domestic violence actually explain this variation almost in its entirety. Thus, 75% or higher prevalence of physical domestic VAC in the community increased the likelihood of this violence across genders (8 times for boys and 6 times for girls). The high magnitude of this effect of community level physical domestic VAC on individual boys and girls, is noteworthy. This finding is in line with social norms (40) and social learning theory (41). According to social norms theory an individual in a particular social gendered context learns to define, imitate, and receive reinforcement for his/her behaviors from the larger society/community (42). As Hall suggests violence is a socially learned behaviour and individuals exposed to violence are more likely to perpetrate it. This highlights the importance of addressing social norms around domestic VAC in the community, which are largely contributing to physical domestic violence against both boys and girls.

This study suffers from some limitations. Violence always tends to be underreported and VAC reported by children is no exception (43). Underreporting may vary by gender, which may introduce measurement errors and compromise comparability across gender. It is noteworthy, that our study is one of the very few studies in Bangladesh that collecting data on VAC directly from the children. This is a strength of our study since there is evidence that underreporting of VAC is likely to be higher when data are collected from the parents (44). Our study carefully followed strategies for enhancing disclosure of violence by ensuring confidentiality, taking interviews in private and in a non-judgemental manner using validated standard tools. The data on lifetime physical domestic VAC were collected retrospectively in this study, which raises concerns regarding recall bias.

This analysis included both primary and secondary data. Consequently, the choice of covariates was constrained by the availability of information. However, strengths of this study include more in-depth and nuanced understanding of how different factors predict physical domestic violence against boys and girls. The findings clearly demonstrate that the same factor may have different effect on the outcome when separate models are run for boys and girls. This finding highlights that it is critical to conduct gender segregated analyses of predictors of VAC so that the nuances introduced by gender can be captured. Methodological strengths of this study include as well use of longitudinal data and the careful choice of covariates, paying attention to the temporality of the events. Moreover, the use of multi-level modeling enabled us to come up with robust estimates and allowed us to explain the variations to a large extent. Further, findings from this study indicate that in this setting with very widespread physical domestic violence among boys and girls, it is absolutely necessary to address violence conducive social norms so that VAC in the home is reduced. It is also important to address gender inequality and ensure equal treatment for both boys and girls.

## Data availability statement

The raw data supporting the conclusions of this article will be made available by the authors, without undue reservation.

## Ethics statement

The studies involving humans were approved by icddr,b’s institutional review board and Swedish Research Ethics Authority. The studies were conducted in accordance with the local legislation and institutional requirements. Written informed consent for participation was not required from the participants or the participants' legal guardians/next of kin because we collected data on respondents violence. We did not want to keep any signature that can identify the respondents. Therefore we took oral consent.

## Author contributions

The study was conceived by RTN and designed by RTN and KP. RTN, JFA, KP, and SZ contributed to model construction. The data were analysed by JFA under the guidance of RTN. The manuscript was drafted by RTN, JFA, and KP and critically reviewed for important intellectual content by all authors. JFA attests that all listed authors meet authorship criteria and that no others meeting the criteria have been omitted. The guarantor (RTN) accepts full responsibility for the work, she accessed the data, and controlled the decision to publish. All authors contributed to the article and approved the submitted version.
