# Supplementary material for: Multi-level analysis of the determinants of physical domestic violence against children using longitudinal data from MINIMat mother–child cohort in Bangladesh
Source: Front Public Health. 2023 Dec 29;11:1185130. doi: 10.3389/fpubh.2023.1185130 (PMC10785797; doi:10.3389/fpubh.2023.1185130)
Supplement: Supplementary file 1 [file Data_Sheet_1.docx]

Appendix Table 1. Validated scale for measuring physical domestic violence against children in a sub-district of Bangladesh

| **Items retained** |
| --- |
| 1. Threatened to hurt or kill you? |
| 2. Shook you aggressively? |
| 3. Slapped you on the face or on back of head? |
| 4. Hit you on the buttocks with an object (such as a stick, broom, cane, or belt)? |
| 5. Hit you over and over again with object or fist (“beat-up”)? |
| 6. Choked you to prevent you from breathing? |
| 7. Twisted your ear? |
| 8. Pulled your hair? |
